# Supplementary material for: Analysing factors influencing railway accidents: A predictive approach using multinomial logistic regression and data mining
Source: PLoS One. 2025 Oct 7;20(10):e0333876. doi: 10.1371/journal.pone.0333876 (PMC12503324; doi:10.1371/journal.pone.0333876)
Supplement: S1 File — (DOCX) [file pone.0333876.s001.docx]

**Annex A: Logistic regression model**

| **Parameter Estimates** | | | | | | | | | | | | | | |  |
| --- | --- | --- | --- | --- | --- | --- | --- | --- | --- | --- | --- | --- | --- | --- | --- |
| **no of accidents category** | | **B  (SE)** | **Wald** | **df** | **Sig.** | **Adjusted OR = Exp(B)** | **95% CI** | **no of accidents category** | | **B (SE)** | **Wald** | **df** | **Sig.** | **Adjusted OR = Exp(B)** | **95% CI** |
| 2 | Intercept | 31.585 (12.599) | 0 | 1 | 0.998 |  |  | 3 | Intercept | -68.938 (12.409) | 0 | 1 | 0.996 |  |  |
|  | month_7=0 | -0.632  (2.082) | 0 | 1 | 1 | 1.88E+03 | [-4.71, 3.45] |  | month_7=0 | -30.609 (2.055) | 0 | 1 | 0.988 | 1.96E+16 | [-34.64, -26.58] |
|  | interest_rates_cat=1 | -32.896 (6.211) | 0 | 1 | 0.996 | 5.17E-12 | [-45.07, -20.73] |  | interest_rates_cat=1 | 13.594 (8.380) | 0 | 1 | 0.999 | 8.01E+08 | [-2.83, 30.02] |
|  | interest_rates_cat=2 | -33.634 (6.685) | 0 | 1 | 0.996 | 2.47E-12 | [-46.74, -20.53] |  | interest_rates_cat=2 | -4.082  (9.692) | 0 | 1 | 1 | 2.017 | [-23.08, 14.91] |
|  | interest_rates_cat=3 | -34.137 (7.274) | 0 | 1 | 0.996 | 1.49E-12 | [-48.40, -19.87] |  | interest_rates_cat=3 | -17.934 (10.508) | 0 | 1 | 0.999 | 1.63E-05 | [-38.53, 2.66] |
|  | interest_rates_cat=4 | -65.323 (6.577) | 0 | 1 | 0.992 | 4.27E-26 | [-78.22, -52.43] |  | interest_rates_cat=4 | 11.321 (8.603) | 0 | 1 | 0.999 | 82.541.078 | [-5.54, 28.18] |
|  | interest_rates_cat=5 | -33.475 (6.219) | 0 | 1 | 0.996 | 2.90E-12 | [-45.66, -21.29] |  | interest_rates_cat=5 | -3.510  (8.963) | 0 | 1 | 1 | 0.03 | [-21.07, 14.05] |
|  | interest_rates_cat=6 | -34.137 (5.376) | 0 | 1 | 0.995 | 1.49E-12 | [-44.67, -23.60] |  | interest_rates_cat=6 | -17.934 (7.766) | 0 | 1 | 0.998 | 1.63E-05 | [-33.15, -2.72] |
|  | interest_rates_cat=7 | -16.589 (7.057) | 0 | 1 | 0.998 | 6.25E-05 | [-30.42, -2.76] |  | interest_rates_cat=7 | -2.336 (10.352) | 0 | 1 | 1 | 0.097 | [-22.63, 17.96] |
|  | interest_rates_cat=8 | -16.931 (6.413) | 0 | 1 | 0.998 | 4.44E-05 | [-29.50, -4.36] |  | interest_rates_cat=8 | 29.419 (8.719) | 0 | 1 | 0.997 | 5.98E+15 | [12.33, 46.51] |
|  | interest_rates_cat=9 | -16.589 (7.057) | 0 | 1 | 0.998 | 6.25E-05 | [-30.42, -2.76] |  | interest_rates_cat=9 | -2.336 (10.352) | 0 | 1 | 1 | 0.097 | [-22.63, 17.96] |
|  | interest_rates_cat=10 | -33.444 (5.376) | 0 | 1 | 0.995 | 2.99E-12 | [-43.99, -22.90] |  | interest_rates_cat=10 | -18.258 (8.514) | 0 | 1 | 0.998 | 1.18E-05 | [-34.95, -1.57] |
|  | interest_rates_cat=11 | -16.589 (7.057) | 0 | 1 | 0.998 | 6.25E-05 | [-30.42, -2.76] |  | interest_rates_cat=11 | -2.336 (10.352) | 0 | 1 | 1 | 0.097 | [-22.63, 17.96] |
|  | interest_rates_cat=12 | -50.664 (7.051) | 0 | 1 | 0.994 | 9.93E-20 | [-64.48, -36.85] |  | interest_rates_cat=12 | -18.623 (8.741) | 0 | 1 | 0.998 | 8.17E-06 | [-35.76, -1.49] |
|  | interest_rates_cat=13 | -49.836 (9.654) | 0 | 1 | 0.996 | 2.27E-19 | [-68.76, -30.91] |  | interest_rates_cat=13 | 14.168 (10.531) | 0 | 1 | 0.999 | 1.42E+09 | [-6.47, 34.81] |
|  | interest_rates_cat=14 | -50.416 (6.841) | 0 | 1 | 0.994 | 1.27E-19 | [-63.83, -37.00] |  | interest_rates_cat=14 | 42.010 (8.940) | 0 | 1 | 0.996 | 1.76E+21 | [24.49, 59.53] |
|  | interest_rates_cat=16 | -50.580 (9.091) | 0 | 1 | 0.996 | 1.08E-19 | [-68.40, -32.76] |  | interest_rates_cat=16 | 40.426 (12.301) | 0 | 1 | 0.997 | 3.60E+20 | [16.32, 64.53] |
|  | interest_rates_cat=17 | -34.506 (6.555) | 0 | 1 | 0.996 | 1.03E-12 | [-47.35, -21.66] |  | interest_rates_cat=17 | 11.984 (8.992) | 0 | 1 | 0.999 | 1.60E+08 | [-5.64, 29.61] |
|  | interest_rates_cat=19 | -17.629 (7.551) | 0 | 1 | 0.998 | 2.21E-05 | [-32.43, -2.83] |  | interest_rates_cat=19 | 13.241 (10.062) | 0 | 1 | 0.999 | 5.63E+08 | [-6.48, 32.97] |
|  | interest_rates_cat=20 | -50.580 (9.091) | 0 | 1 | 0.996 | 1.08E-19 | [-68.40, -32.76] |  | interest_rates_cat=20 | 40.426 (12.301) | 0 | 1 | 0.997 | 3.60E+20 | [16.32, 64.53] |
|  | interest_rates_cat=21 | -33.634 (7.426) | 0 | 1 | 0.996 | 2.47E-12 | [-48.19, -19.08] |  | interest_rates_cat=21 | -4.082 (10.759) | 0 | 1 | 1 | 0.02 | [-25.17, 17.01] |
|  | interest_rates_cat=22 | -33.666 (12.855) | 0 | 1 | 0.998 | 2.39E-12 | [-58.86, -8.47] |  | interest_rates_cat=22 | 32.671 (13.020) | 0 | 1 | 0.998 | 1.54E+17 | [7.15, 58.19] |
|  | interest_rates_cat=23 | -32.792 (12.922) | 0 | 1 | 0.998 | 5.74E-12 | [-58.12, -7.46] |  | interest_rates_cat=23 | 15.914 (13.514) | 0 | 1 | 0.999 | 8.15E+09 | [-10.59, 42.42] |
|  | interest_rates_cat=24 | -32.792 (12.117) | 0 | 1 | 0.998 | 5.74E-12 | [-56.54, -9.04] |  | interest_rates_cat=24 | 15.914 (11.878) | 0 | 1 | 0.999 | 8.15E+09 | [-7.38, 39.21] |
|  | interest_rates_cat=25 | -32.420 (12.996) | 0 | 1 | 0.998 | 8.32E-12 | [-57.89, -6.95] |  | interest_rates_cat=25 | 17.476 (13.419) | 0 | 1 | 0.999 | 3.89E+10 | [-8.83, 43.78] |
|  | interest_rates_cat=26 | -32.792 (12.117) | 0 | 1 | 0.998 | 5.74E-12 | [-56.54, -9.04] |  | interest_rates_cat=26 | 15.914 (11.878) | 0 | 1 | 0.999 | 8.15E+09 | [-7.38, 39.21] |
|  | interest_rates_cat=27 | -32.792 (14.508) | 0 | 1 | 0.998 | 5.74E-12 | [-61.23, -4.36] |  | interest_rates_cat=27 | 15.914 (14.893) | 0 | 1 | 0.999 | 8.15E+09 | [-13.28, 45.11] |
|  | interest_rates_cat=28 | -66.929 (13.397) | 0 | 1 | 0.996 | 8.58E-27 | [-93.19, -40.67] |  | interest_rates_cat=28 | -2.020 (13.880) | 0 | 1 | 1 | 0.133 | [-29.23, 25.19] |
|  | interest_rates_cat=29 | -48.994 (16.566) | 0 | 1 | 0.998 | 5.27E-19 | [-81.46, -16.53] |  | interest_rates_cat=29 | 34.164 (15.439) | 0 | 1 | 0.998 | 6.87E+17 | [3.90, 64.43] |
|  | interest_rates_cat=30 | -0.031 (12.751) | 0 | 1 | 1 | 0.97 | [-25.02, 24.96] |  | interest_rates_cat=30 | 36.062 (12.578) | 0 | 1 | 0.998 | 4.59E+18 | [11.41, 60.72] |
|  | interest_rates_cat=31 | -15.361 (16.486) | 0 | 1 | 0.999 | 2.13E-04 | [-47.68, 16.96] |  | interest_rates_cat=31 | 38.246 (15.067) | 0 | 1 | 0.998 | 4.07E+19 | [8.72, 67.77] |
|  | interest_rates_cat=32 | -32.160 (14.656) | 0 | 1 | 0.998 | 1.08E-11 | [-61.00, -3.32] |  | interest_rates_cat=32 | 46.523 (15.034) | 0 | 1 | 0.998 | 1.60E+23 | [17.06, 75.99] |
|  | interest_rates_cat=33 | -66.929 (14.886) | 0 | 1 | 0.996 | 8.58E-27 | [-96.11, -37.75] |  | interest_rates_cat=33 | -2.020 (15.653) | 0 | 1 | 1 | 0.133 | [-32.70, 28.66] |
|  | interest_rates_cat=34 | -15.361 (13.220) | 0 | 1 | 0.999 | 2.13E-04 | [-41.27, 10.55] |  | interest_rates_cat=34 | 38.246 (13.895) | 0 | 1 | 0.998 | 4.07E+19 | [11.01, 65.49] |
|  | interest_rates_cat=35 | -15.361 (14.588) | 0 | 1 | 0.999 | 2.13E-04 | [-43.95, 13.23] |  | interest_rates_cat=35 | 38.246 (13.720) | 0 | 1 | 0.998 | 4.07E+19 | [11.36, 65.13] |
|  | interest_rates_cat=36 | -66.929 (14.338) | 0 | 1 | 0.996 | 8.58E-27 | [-95.13, -38.73] |  | interest_rates_cat=36 | -2.020 (14.546) | 0 | 1 | 1 | 0.133 | [-30.53, 26.49] |
|  | interest_rates_cat=37 | -16.234 (12.751) | 0 | 1 | 0.999 | 8.91E-05 | [-41.22, 8.75] |  | interest_rates_cat=37 | 54.311 (12.578) | 0 | 1 | 0.997 | 3.87E+26 | [29.66, 78.96] |
|  | interest_rates_cat=39 | -15.361 (12.819) | 0 | 1 | 0.999 | 2.13E-04 | [-40.49, 9.77] |  | interest_rates_cat=39 | 38.246 (13.087) | 0 | 1 | 0.998 | 4.07E+19 | [12.59, 63.90] |
|  | interest_rates_cat=40 | -49.498 (13.634) | 0 | 1 | 0.997 | 3.19E-19 | [-76.22, -22.78] |  | interest_rates_cat=40 | 20.312 (14.707) | 0 | 1 | 0.999 | 6.62E+11 | [-8.52, 49.14] |
|  | interest_rates_cat=41 | -15.228 (12.562) | 0 | 1 | 0.999 | 2.44E-04 | [-39.85, 9.39] |  | interest_rates_cat=41 | 38.719 (12.515) | 0 | 1 | 0.998 | 6.54E+19 | [14.19, 63.25] |
|  | interest_rates_cat=42 | -49.498 (13.634) | 0 | 1 | 0.997 | 3.19E-19 | [-76.22, -22.78] |  | interest_rates_cat=42 | 20.312 (14.707) | 0 | 1 | 0.999 | 6.62E+11 | [-8.52, 49.14] |
|  | interest_rates_cat=43 | -16.203 (11.782) | 0 | 1 | 0.999 | 9.19E-05 | [-39.30, 6.89] |  | interest_rates_cat=43 | 18.250 (11.006) | 0 | 1 | 0.999 | 8.43E+10 | [-3.32, 39.82] |
|  | interest_rates_cat=45 | -33.506 (7.566) | 0 | 1 | 0.996 | 2.81E-12 | [-48.34, -18.67] |  | interest_rates_cat=45 | 12.675 (10.708) | 0 | 1 | 0.999 | 3.20E+08 | [-8.32, 33.67] |
|  | interest_rates_cat=47 | -16.203 (11.782) | 0 | 1 | 0.999 | 9.19E-05 | [-39.30, 6.89] |  | interest_rates_cat=47 | 18.250 (11.006) | 0 | 1 | 0.999 | 8.43E+10 | [-3.32, 39.82] |
|  | interest_rates_cat=49 | -34.137 (7.274) | 0 | 1 | 0.996 | 1.49E-12 | [-48.40, -19.87] |  | interest_rates_cat=49 | -17.934 (10.508) | 0 | 1 | 0.999 | 1.63E-05 | [-38.53, 2.66] |
|  | fertility rate_cat=4 | 17.431 (7.184) | 0 | 1 | 0.998 | 3.72E+10 | [3.35, 31.51] |  | fertility rate_cat=4 | 22.332 (8.337) | 0 | 1 | 0.998 | 5.00E+12 | [6.00, 38.66] |
|  | fertility rate_cat=5 | 33.634 (3.653) | 0 | 1 | 0.993 | 4.04E+17 | [26.47, 40.80] |  | fertility rate_cat=5 | 4.082  (5.341) | 0 | 1 | 0.999 | 5.93E+04 | [-6.39, 14.55] |
|  | mariage rate_cat=1 | -32.406 (11.297) | 0 | 1 | 0.998 | 8.44E-12 | [-54.55, -10.26] |  | mariage rate_cat=1 | 36.499 (9.862) | 0 | 1 | 0.997 | 7.10E+18 | [17.17, 55.83] |
|  | mariage rate_cat=2 | -15.361 (11.531) | 0 | 1 | 0.999 | 2.13E-04 | [-37.96, 7.24] |  | mariage rate_cat=2 | 38.246 (10.288) | 0 | 1 | 0.997 | 4.07E+19 | [18.08, 58.41] |
|  | mariage rate_cat=3 | -32.552 (12.221) | 0 | 1 | 0.998 | 7.30E-12 | [-56.50, -8.60] |  | mariage rate_cat=3 | -24.196 (10.872) | 0 | 1 | 0.998 | 3.10E-08 | [-45.50, -2.89] |
|  | mariage rate_cat=5 | -16.203 (0.000) | . | 1 | . | 9.19E-05 | [-16.20, -16.20] |  | mariage rate_cat=5 | 18.250 (0.000) | . | 1 | . | 8.43E+10 | [18.25, 18.25] |
