# Supplementary material for: Analysing factors influencing railway accidents: A predictive approach using multinomial logistic regression and data mining
Source: PLoS One. 2025 Oct 7;20(10):e0333876. doi: 10.1371/journal.pone.0333876 (PMC12503324; doi:10.1371/journal.pone.0333876)
Supplement: Attachment — Submitted filename: response_rev1.docx [file pone.0333876.s002.docx]

# Response for Reviewer 1

Dear Reviewer,

Thank you very much for your effort and time spent on our paper. Based on your comments and other reviewers’ comments, we did our best to improve the quality of our paper. Please let us respond to your comments in this letter.

Comment 1: Graphical representation needed.

Response 1: We added the graphical representation of the distributions of the input variables with respect to the categories of the outcome variable accident rate. Moreover, we added a graphical representation of the model’s prediction performance through the ROC curves. We hope these graphs will help the readers of the paper better understand the distribution of the data used in the study and its results. If you have any other graphs, you consider suitable for inclusion in the paper, kindly please let us know, and we will do our best to create and incorporate them. Thank you for the suggestion.

Comment 2: Age, sex, demographic data missing.

Response 2: Thank you for your valuable feedback and for suggesting the inclusion of additional variables, such as demographic characteristics, in our analysis. However, in our study, the data cases represent not the individual records of railway accidents but rather the individual months of the period under review, along with their corresponding accident rates. In more detail, we have aggregated microdata on railway accidents into a monthly total of accidents. Then, when creating prediction models, we categorised this total into three levels of accident rate: lower, middle, and higher. Since we are working with aggregated data rather than individual accident records, we do not use specific demographic details of the accident participants. Consequently, it is not feasible to incorporate the demographic characteristics of the accident participants, such as age and gender, directly into our predictive model. Instead, we focused the analysis on socio-economic factors characterising broader societal and economic conditions, which may indirectly influence railway accident rates. We believe this approach aligns with the scope and objectives of our study.

We hope this explanation clarifies our methodological choices. In our paper, we have expanded the discussion section to explain the selection of the variables for our analysis in more detail and, besides that, to clarify the non-selection of other potential factors influencing railway safety and accidents of the mentioned type.

Comment 3: The present study should become a base for further study on prevention of such accidents, so kindly highlight the steps taken by the authors corresponding to railway officials in the discussion part.

Response 3: The issue of this type of rail suicide and its prevention is highly complex. Several particularly effective measures can help reduce the number of suicides on the railways. These measures typically involve a combination of prevention, awareness-raising, technical solutions, and mental health support. Some examples are as follows:

- Analysis of Patterns and Data: Data on suicides and attempted suicides can be utilised to identify areas and times of highest risk. Based on this data, railway companies can optimise their response and concentrate on areas that require immediate attention.

- Cameras and sensors: The most critical points should be monitored by cameras and sensors, immediately identifying individuals in danger and allowing for immediate reaction.

- Physical barriers: There are examples from other countries of installing protective barriers at critical points on railway infrastructure. These barriers can prevent people from entering the tracks.

All three measures mentioned are strongly connected to the analyses of historical records of railway incidents to identify the most critical points on the railways or periods with the highest risk. Therefore, analytical studies, such as those presented in our paper, are of high importance, allowing for employing evidence-based protective measures.

Another viewpoint on the protective measures is providing mental health support and intervention. This topic is of great importance and should be a priority for the government to support the organisations providing mental health support, consultancy, and crisis lines. Critical locations on rail infrastructure can be equipped with tables displaying emergency contact telephone numbers.

Last but not least, based on an analysis of critical locations and times of accidents, it is possible to identify an increased likelihood of an accident situation and alert railway staff. Employees such as drivers, conductors and station staff should be trained to identify risk situations and provide first aid in crisis situations. They should also know how to communicate effectively with individuals in psychological distress and guide them to the appropriate help.

In any case, all these measures require long-term cooperation between railway companies, organisations providing mental health support, and policymakers.

We have added these potential measures to the discussion part of the paper.
