# Supplementary material for: Analysing factors influencing railway accidents: A predictive approach using multinomial logistic regression and data mining
Source: PLoS One. 2025 Oct 7;20(10):e0333876. doi: 10.1371/journal.pone.0333876 (PMC12503324; doi:10.1371/journal.pone.0333876)
Supplement: Attachment — Submitted filename: Comments.docx [file pone.0333876.s003.docx]

**Previous Comments**

**Resolved Reviewer Comments**

1. **Clarity of Objective and Rationale**
   - **Resolved**: The study's objectives are now clearly articulated, and the rationale for using socio-economic factors is well-grounded in both the introduction and the discussion. The revision demonstrates improved alignment between the research aim, theoretical background, and methodology.
2. **Data Sources and Variables Justification**
   - **Resolved**: Authors have added a comprehensive explanation of each variable, including sources (Table 4), relevance, and limitations due to data availability. This adequately addresses earlier concerns regarding variable selection.
3. **Model Development Process**
   - **Resolved**: The use of the CRISP-DM framework is now clearly explained. The rationale behind binning and the transformation from regression to classification (multinomial logistic regression) is well justified.
4. **Interpretation of Key Predictors**
   - **Resolved**: Authors provide a detailed interpretation of key variables (e.g., interest rate, marriage rate, fertility rate), and integrate relevant literature in the discussion.
5. **Policy Implications**
   - **Resolved**: Practical applications of the model, particularly for suicide prevention and rail safety measures, are now described in sufficient depth.

**Partially Resolved Reviewer Comments**

1. **Reporting of Effect Sizes / Regression Coefficients**
   - **Partially Resolved**: While some interpretation is offered, the manuscript **does not provide adjusted odds ratios or confidence intervals** in tabular form for transparency and interpretability. This limits understanding of the magnitude and reliability of associations.
2. **COVID-19 Pandemic Influence**
   - **Partially Resolved**: The authors included a binary “COVID” variable and provide some descriptive comparisons, but a more nuanced temporal analysis (e.g., trend interruptions, seasonal adjustments) was not attempted, which could have enriched the findings.

New comment

1. **Model Validation and Overfitting**

- The predictive performance of the model is notably high (accuracy > 92%, Nagelkerke R² = 0.964), raising concerns of potential overfitting, especially given the categorisation of both the dependent and independent variables.
- **Recommendation**: Include internal validation techniques such as k-fold cross-validation or bootstrapping. Discuss generalisability and any steps taken to prevent overfitting.
