# Supplementary material for: Analysing factors influencing railway accidents: A predictive approach using multinomial logistic regression and data mining
Source: PLoS One. 2025 Oct 7;20(10):e0333876. doi: 10.1371/journal.pone.0333876 (PMC12503324; doi:10.1371/journal.pone.0333876)
Supplement: Attachment — Submitted filename: response_rev5_2.docx [file pone.0333876.s004.docx]

# Response for Reviewer 5

Dear Reviewer,

Thank you very much for your effort and time spent on our paper. Based on your comments and other reviewers’ comments, we did our best to improve the quality of our paper. Please allow us to respond to your comments in this letter.

Comment 1: While some interpretation is offered, the manuscript does not provide adjusted odds ratios or confidence intervals in tabular form for transparency and interpretability. This limits understanding of the magnitude and reliability of associations.

Response 1: Thank you for your comment. The entire table with the coefficients and their significance tests is in Annex A. We have slightly restructured this table by combining the Beta coefficients and their Standard Errors (SE) into one column. Additionally, we have included the 95% confidence intervals (CI) in the last column of the table.

Comment 2: The authors included a binary “COVID” variable and provide some descriptive comparisons, but a more nuanced temporal analysis (e.g., trend interruptions, seasonal adjustments) was not attempted, which could have enriched the findings.

Response 2: Thank you for the suggestions. Please let us clarify our approach. Our outcome variable consists of accident counts aggregated monthly and then categorised into three accident rate levels (low, middle, and high). Each monthly observation is considered independent, as there is no evidence that the occurrence of suicides in one month directly depends on the previous month. To reinforce this point, we checked the dependence of accident numbers using autocorrelations and partial autocorrelations; please see the following figures.

|  |  |
| --- | --- |

The blue columns in the figures show the strength of the correlation between the number of accidents and its individual lags, with the lags of one to 24 months depicted. The black horizontal lines indicate the bounds for the significance tests of the correlations. As can be seen, all the correlations were statistically insignificant.

Furthermore, we performed the Box-Ljung test to determine whether the time series of accident numbers exhibits a white noise pattern, meaning they are uncorrelated and lack significant serial autocorrelation. The null hypothesis states that there is no serial correlation (i.e., the series is white noise), while the alternative hypothesis suggests there is significant serial correlation. A significant p-value (usually < 0.05) leads to rejecting the null hypothesis, indicating the series is not white noise, whereas p-values higher than the 0.05 significance level imply we cannot reject the null hypothesis. The test results are presented in the following table.

Based on these results (the last column shows the p-values), we can treat the series of accident numbers as white noise. Therefore, including its lags in the regression model is unnecessary, as they would also be insignificant. In conclusion, we tested for serial correlation and found no evidence of dependence; thus, it is unnecessary to use a time series model with autocorrelations for modelling this series.

Apart from that, the strongest argument against using classical linear regression is that our outcome variable is not quantitative. Instead, it is categorical (accident rate categories). Therefore, conventional linear regression methods cannot be applied, nor can linear time series models like ARIMA. Consequently, a traditional regression approach was unsuitable, and logistic regression was the appropriate choice. Additionally, we agree with the reviewer that it is interesting to investigate how specific seasons influence accident numbers and how they are developing over time. To examine that, we included dummy variables for individual months and the four seasons of the year into the model and a year as a time component of the model. Therefore, our final choice was a logistic regression model with temporal predictors, such as month and season dummies and time component represented by a year.

Of course, since we used the forward stepwise method for model creation, not all the variables mentioned above appeared in the final model (see Annex A in the paper). On the other hand, we recognise that there are several more sophisticated and complex methods to model this particular case. Nonetheless, their main disadvantage is often their poor readability and difficulty in interpretation. Therefore, we consider that using logistic regression is suitable for this specific situation.

Comment 3: The predictive performance of the model is notably high (accuracy > 92%, Nagelkerke R² = 0.964), raising concerns of potential overfitting, especially given the categorisation of both the dependent and independent variables. Include internal validation techniques such as k-fold cross-validation or bootstrapping. Discuss generalisability and any steps taken to prevent overfitting.

Response 3: Dear Reviewer, as mentioned in the Methodology section, to avoid overfitting, we divided our data into a training set and a testing set, using a ratio of 75% : 25%. The first, the training set, is used for model creation, while the remaining data forms the testing set and is used for model evaluation. It is true that the Nagelkerke R-square is listed for the training set, as it describes the model created on this data. For this reason, such evaluation metrics should suffer from overestimation. However, the confusion matrix presented in the paper shows the model’s performance on the testing data, which was unknown to the model during its creation. The same is true for the ROC curves presented in the paper. Therefore, the evaluation metrics, such as sensitivity or overall accuracy, and the values of AUC are reported in the paper only for the testing data, not for the training data. We agree this may cause some confusion, but we chose this presentation with the best intentions. If you consider it necessary, we can expand the confusion matrix to include the model’s performance on both datasets.

Regarding the bootstrapping method or k-folds cross-validation techniques, we regret so, but it is not possible to perform these methods in the SPSS Statistics and SPSS Modeler software we use for the statistical and data mining analyses. We tried our best to perform at least one of them, but both failed due to their computational complexity. Logistic regression, as you probably know, is a method where coefficients are estimated numerically, which often leads to computational complexity and frequent failures in their estimation. For this reason, bootstrapping did not produce results for the confidence intervals of the coefficient estimates. On the other hand, neither of the two software packages offers the possibility of cross-validation as part of the procedure for multinomial logistic regression (for the binomial case, it would be somewhat possible through manual repetition of model creation k-times, depending on the chosen number of folds).

Nevertheless, we hope that the chosen method of model evaluation by dividing data into training and testing samples, which is a common practice, evaluates the model sufficient. In the paper, we clarify the explanation of the Nagelkerke R-square statistics to prevent confusion.
